# Thyroid Metastasis as the Initial Presentation of Occult Breast Carcinoma: A Case Report

**DOI:** 10.7759/cureus.108770

**Published:** 2026-05-13

**Authors:** Despoina Milonaki, Panagiota Pantoula, Ioannis Provatas, Argyro Fourlopoulou, Nikoleta Sinou

**Affiliations:** 1 Department of General Surgery, General Hospital of Nikaia "Agios Panteleimon", Athens, GRC; 2 Department of Pathology, General Hospital of Nikaia "Agios Panteleimon", Athens, GRC; 3 Department of Public Health Policy, University of West Attica, Athens, GRC; 4 Research and Education in Biomedical Sciences, National and Kapodistrian University of Athens School of Medicine, Athens, GRC

**Keywords:** metastatic breast carcinoma, occult breast cancer, thyroid metastasis, thyroid metastasis to occult breast carcinoma, thyroid nodule

## Abstract

Occult breast cancer (OBC) is a rare clinical entity characterized by metastatic disease in the absence of an identifiable primary breast lesion.

Metastases to the thyroid gland are rare. Breast carcinoma infrequently metastasizes to the thyroid, and cases arising from OBC are exceptionally uncommon, posing a significant diagnostic challenge.

We report the case of a 63-year-old woman with no prior history of malignancy who presented with diffuse abdominal pain, headache, and neck stiffness. A thyroid nodule was identified during diagnostic evaluation. Ultrasonography demonstrated a hypoechoic lesion with irregular margins, and core needle biopsy suggested malignancy. Immunohistochemical (IHC) analysis showed positivity for estrogen receptor (ER), progesterone receptor (PR), mammaglobin, and GATA3, with negativity for thyroglobulin (TG), PAX8, and thyroid transcription factor-1 (TTF-1), supporting a breast origin. Mammography and breast ultrasonography were negative. Breast magnetic resonance imaging (MRI) was not performed due to rapid clinical deterioration. During hospitalization, she developed a generalized tonic-clonic seizure. Imaging revealed multiple brain metastases, indicating advanced disease.

This case underscores the diagnostic complexity of thyroid lesions and highlights the necessity of considering metastatic disease, particularly in the context of OBC. Accurate diagnosis requires comprehensive histopathological assessment supported by immunohistochemical profiling to reliably distinguish metastatic involvement from primary thyroid malignancies. Prompt recognition is crucial to inform optimal therapeutic decision-making in these challenging clinical scenarios.

## Introduction

Metastatic tumors to the thyroid gland represent a rare clinicopathological entity, despite the organ's rich vascularization and high rate of perfusion, second only to the adrenal glands; this paradox is justified by a combination of fast blood circulation preventing tumor cell adhesion and high oxygen saturation and iodine content inhibiting tumor cell growth [1].

Historically, the incidence of thyroid metastases in autopsy series was reported between 1.25% and 24%, yet they remain clinically underestimated, accounting for only 1.4%-3% of all clinical thyroid malignancies [2]. This discrepancy suggests that many thyroid metastases remain asymptomatic or are overshadowed by the progression of the primary disease.

Breast cancer demonstrates a well-established and heterogeneous pattern of metastatic dissemination, with the most common sites being the bones, lung, liver, and brain [3]. Research indicates a clear correlation between molecular subtypes and metastatic tropism: the bone remains the predominant site for hormone receptor-positive disease, whereas visceral and central nervous system involvement is significantly more frequent in HER2-positive and triple-negative breast cancer (TNBC) [3].

In addition to these classic sites, "atypical" metastases to the thyroid, gastrointestinal tract, and spleen have been increasingly reported due to improved diagnostic imaging and longer patient survival. However, thyroid involvement as the initial clinical manifestation of an occult breast carcinoma is an extraordinary rarity. In such cases, the thyroid nodule may be misdiagnosed as a primary thyroid neoplasm, leading to inappropriate surgical management [4]. The "seed and soil" hypothesis suggests that while the thyroid is vascular, its high iodine content and rapid arterial flow may normally inhibit the "seeding" of cancer cells, making a successful metastasis there a sign of aggressive systemic potential [5].

The diagnosis of thyroid metastasis poses significant challenges, as clinical presentations and ultrasonographic findings often mimic primary thyroid malignancies [6]. Therefore, a high index of clinical suspicion is paramount. Beyond imaging, the definitive diagnosis relies on histopathological confirmation through fine-needle aspiration (FNA) or core needle biopsy (CNB). The integration of an expanded immunohistochemical (IHC) panel, specifically markers such as GATA3, mammaglobin, and GCDFP-15 versus thyroid-specific markers such as thyroid transcription factor-1 (TTF-1) and PAX8, is essential for an accurate diagnosis [7].

In this report, we present a rare case of occult breast cancer (OBC) with metastasis to the thyroid gland, highlighting the diagnostic challenges and the importance of considering breast cancer in the differential diagnosis of thyroid lesions. This case underscores the need for a high index of suspicion and individualized management in such atypical presentations.

## Case presentation

A 63-year-old woman presented in September 2025 with progressive generalized malaise, severe headaches, and a six-month history of gradually worsening voice changes. Notably, she had sought medical attention at two separate hospitals during this period; however, no definitive diagnosis was established, and she was discharged with conservative management.

She denied dysphagia, odynophagia, or dyspnea and reported no constitutional symptoms such as weight loss, fever, or night sweats.

Her past medical history was significant for arterial hypertension and type 2 diabetes mellitus. She had no known personal or family history of malignancy.

At the physical examination, a firm, non-tender induration was palpated in the left cervical region. No overlying skin changes or inflammatory signs were noted. There was no clinically evident lymphadenopathy. Cardiopulmonary and abdominal examinations were unremarkable.

Laboratory findings showed that thyroid function tests were within normal limits, and thyroid autoantibodies were negative. Serum tumor markers were largely within normal ranges, with a mild elevation observed in CA 15-3 (Table 1).

**Table 1:** Laboratory findings. TSH, thyroid-stimulating hormone; FT4, free thyroxine; FT3, free triiodothyronine; TPO, thyroid peroxidase; TG, thyroglobulin; CEA, carcinoembryonic antigen

Parameter	Value	Reference Range
TSH	2.1 μIU/mL	0.4-4.0
FT4	1.2 ng/dL	0.8-1.8
FT3	3.1 pg/mL	2.0-4.4
Anti-TPO	12 IU/mL	<35
Anti-TG	18 IU/mL	<40
CEA	2.1 ng/mL	<5
CA 125	18 U/mL	<35
CA 19-9	12 U/mL	<37
CA 15-3	32 U/mL	<30

**Figure 1:**
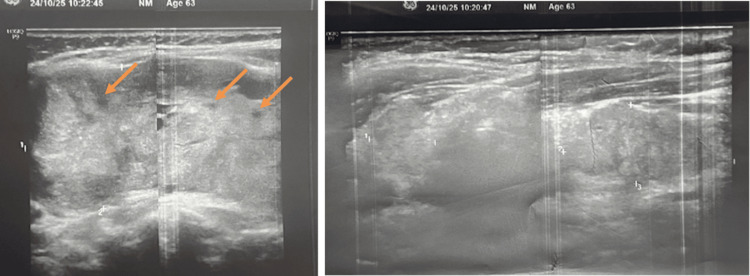
Thyroid ultrasonography demonstrating suspicion for malignancy: multiple small nodules indicated with the arrows.

Thyroid ultrasonography demonstrated a diffusely heterogeneous thyroid parenchyma with multiple small clustered hypoechoic nodules, raising suspicion for malignancy (Thyroid Imaging Reporting and Data System {TIRADS} category 4) (Figure 1).

**Figure 2:**
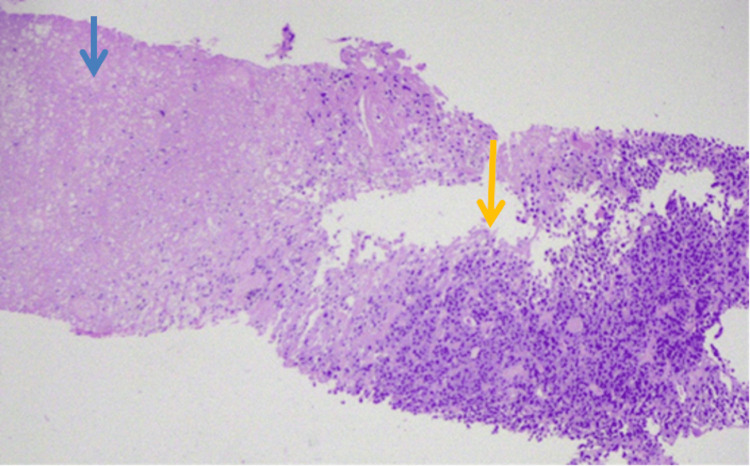
H&E ×10: necrosis (blue arrow) with adjacent viable tumor cells (yellow arrow).

**Figure 3:**
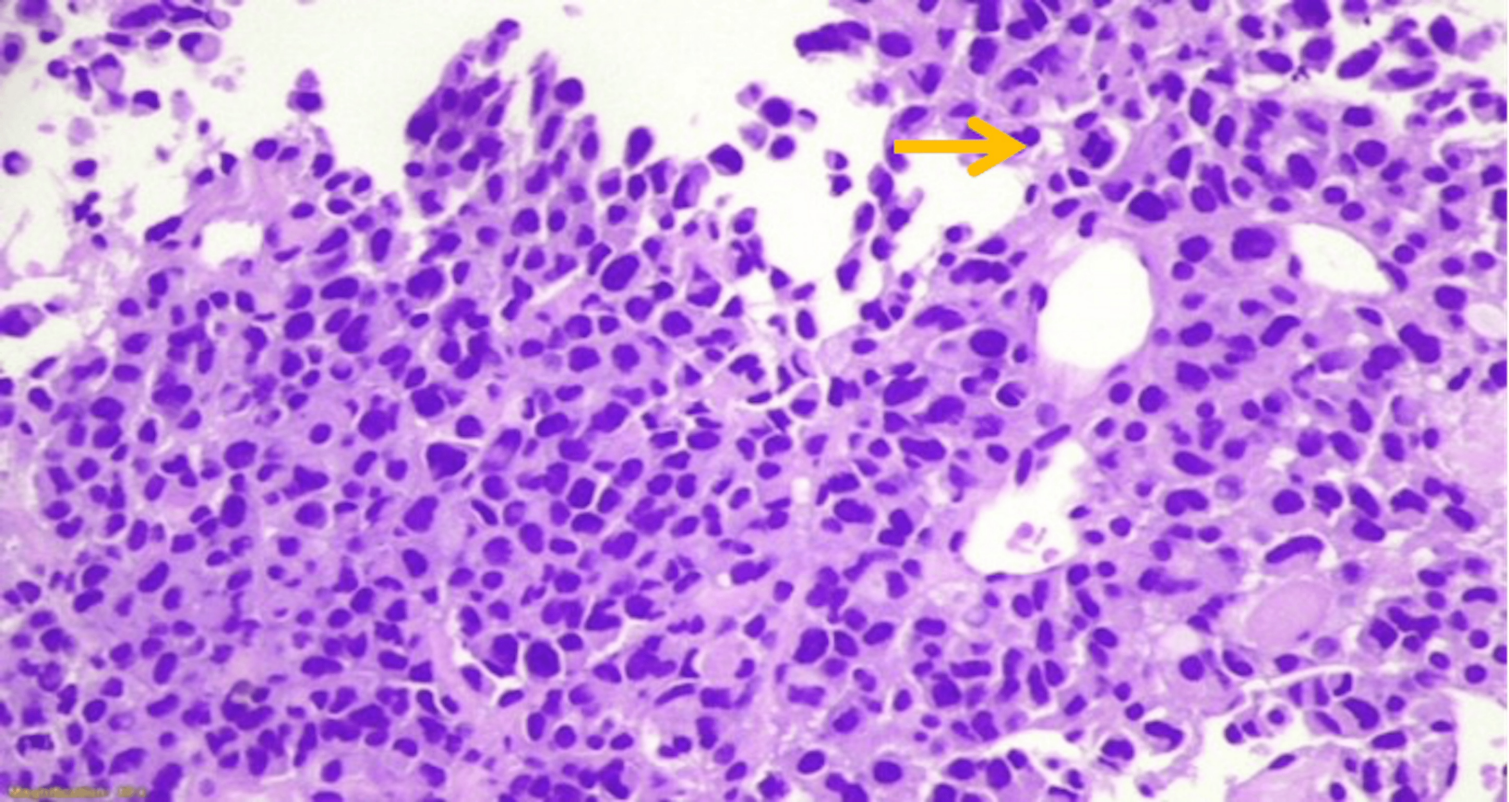
H&E ×10: high-power view demonstrating diffuse infiltration by carcinoma cells with moderate nuclear atypia, eosinophilic cytoplasm, and mitotic activity (yellow arrow).

A fine-needle biopsy (FNB) of the thyroid lesion was subsequently performed. During the histological evaluation, a malignant neoplastic lesion was identified, demonstrating necrosis and diffuse infiltration by carcinoma cells with moderate nuclear atypia, eosinophilic cytoplasm, and mitotic activity (Figures 2, 3).

In order to determine their origin, an extensive immunohistochemical assay was performed, which demonstrated positivity for carcinoembryonic antigen (CEA), estrogen receptors (ER), progesterone receptors (PR), mammaglobin, and GATA3 and negative expression for thyroglobulin (TG), PAX8, and TTF-1, supporting the diagnosis of metastatic breast carcinoma to the thyroid gland.

**Figure 4:**
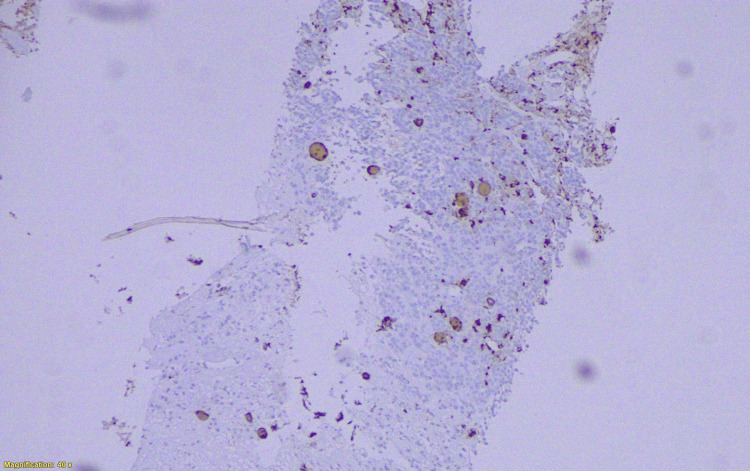
Thyroglobulin (×10): negative staining of tumor cells excludes thyroid origin.

**Figure 5:**
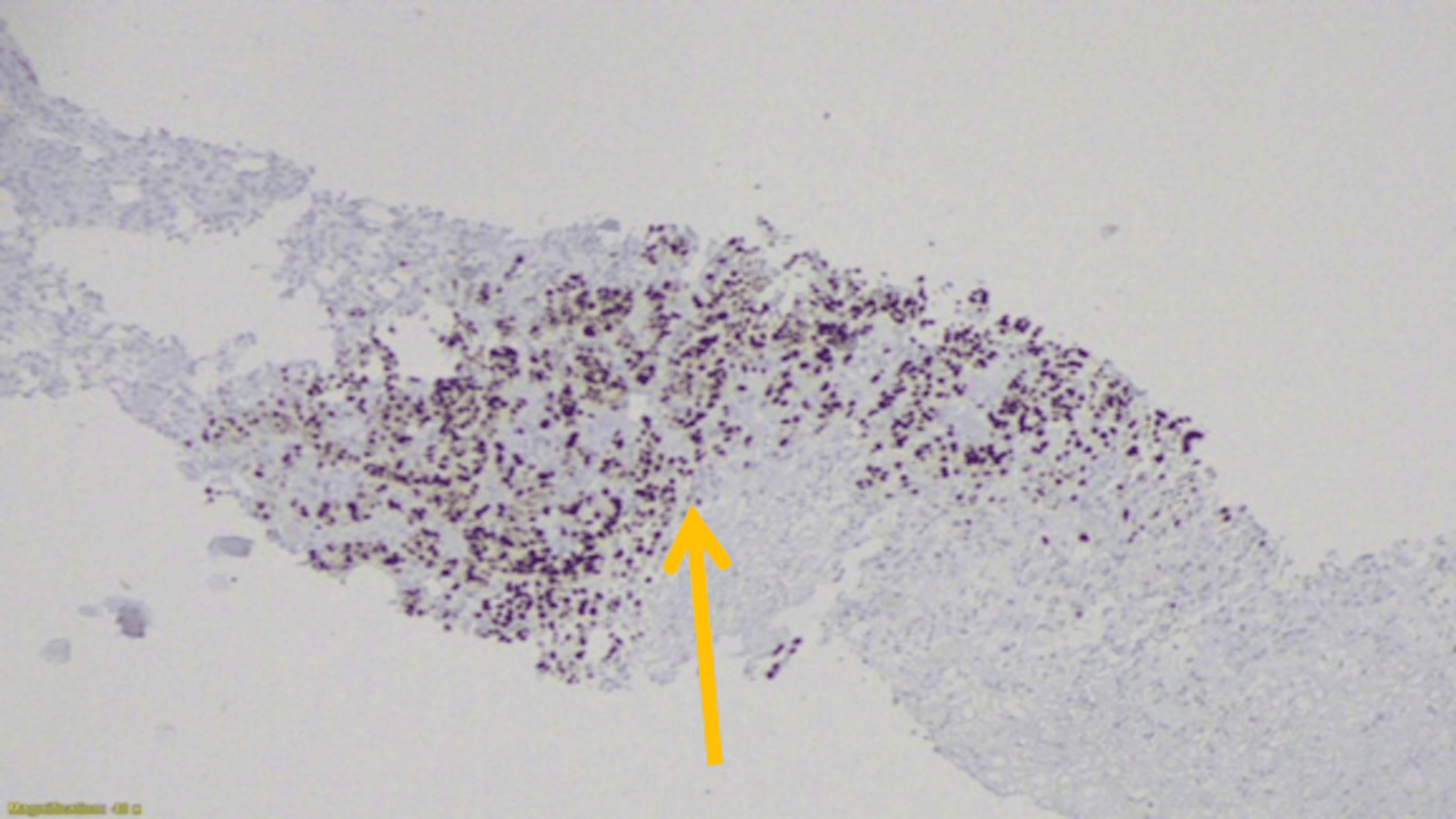
GATA3 (×10): nuclear expression of GATA3 in neoplastic cells.

**Figure 6:**
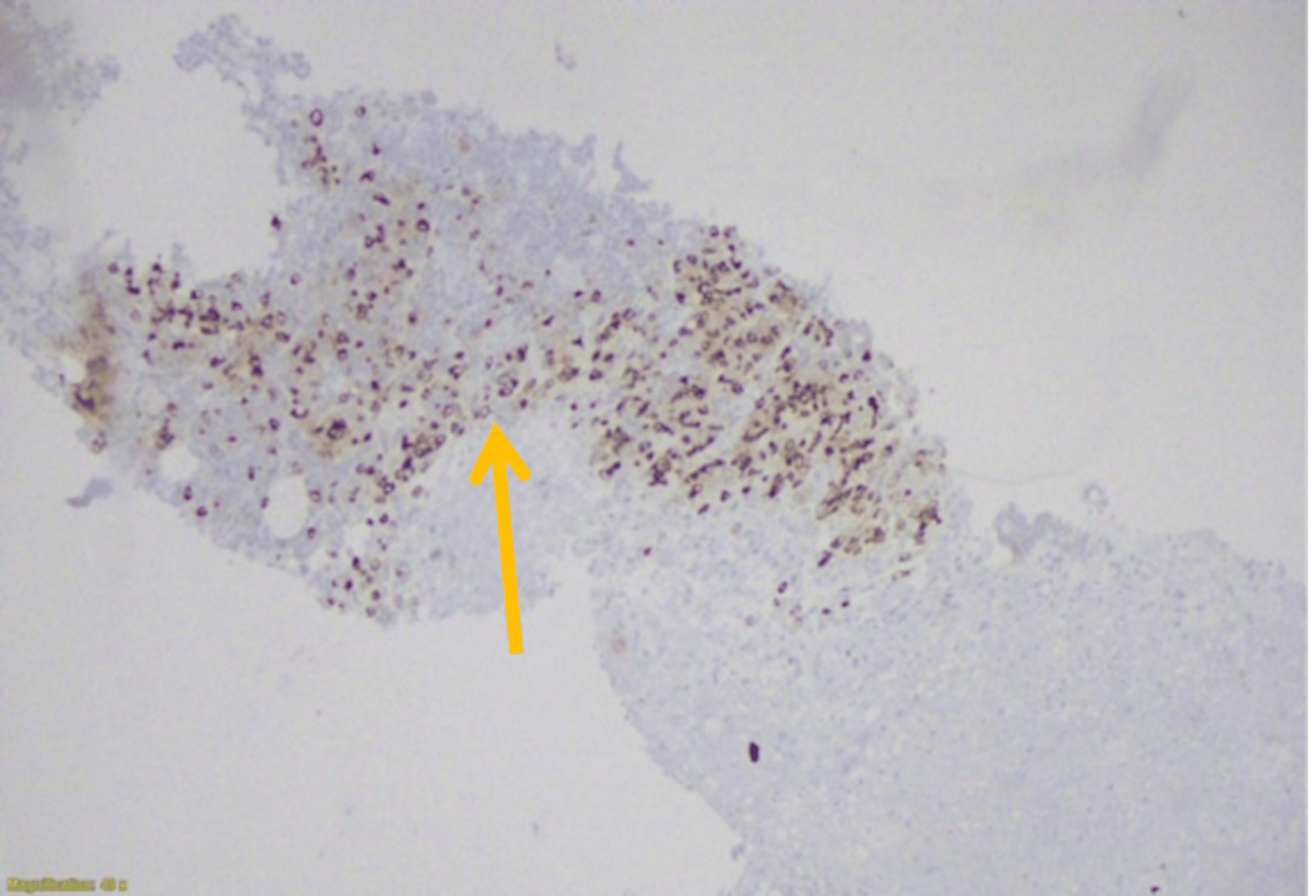
Mammaglobin (×10): moderate to strong cytoplasmic and membranous mammaglobin positivity in a significant subset of carcinoma.

Immunohistochemical analysis demonstrated positivity for mammaglobin, GATA3, estrogen, and progesterone receptors and negativity for thyroglobulin, PAX8, and TTF-1, supporting the diagnosis of metastatic breast carcinoma to the thyroid gland (Figures 4-6).

The immunohistochemical profile is presented in Table 2.

**Table 2:** Immunohistochemistry profile. CEA, carcinoembryonic antigen; ER, estrogen receptor; PR, progesterone receptor; TTF-1, thyroid transcription factor-1

Marker	Result	Interpretation
CEA	Positive	Supports epithelial origin
ER	Positive	Breast origin
PR	Positive	Breast origin
Mammaglobin	Positive	Breast-specific marker
GATA3	Positive	Strongly supports breast carcinoma
Thyroglobulin	Negative	Excludes primary thyroid tumor
PAX8	Negative	Not thyroid origin
TTF-1	Negative	Not lung/thyroid origin
E-cadherin	Negative	Suggests lobular phenotype
HER2	3+	HER2-positive disease

Following this, the further evaluation of the breasts was carried out. No palpable breast masses were identified on clinical examination.

Moreover, no suspected axillary lymphadenopathy was detected, except for some benign-looking lymph nodes. Additionally, both breast ultrasonography and mammography revealed no suspicious or pathological findings. Breast magnetic resonance imaging (MRI) was also not performed, whereas positron emission tomography (PET) was not conducted due to rapid clinical deterioration.

This discordance between metastatic disease and the absence of an identifiable primary breast lesion highlights the diagnostic challenge of occult breast carcinoma.

**Figure 7:**
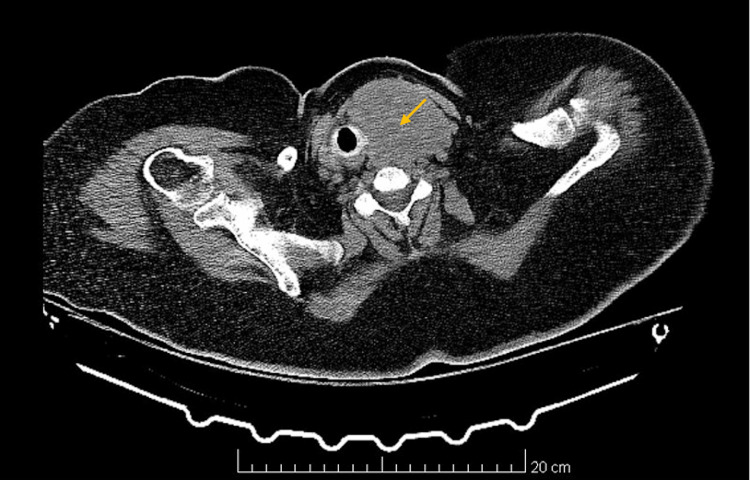
Chest computed tomography: heterogeneous mass in the left cervical region.

A chest CT scan performed for further evaluation revealed a large heterogeneous mass in the left cervical region, extending to the thoracic inlet and measuring 12.2 × 7 cm in the frontal plane (Figure 7).

**Figure 8:**
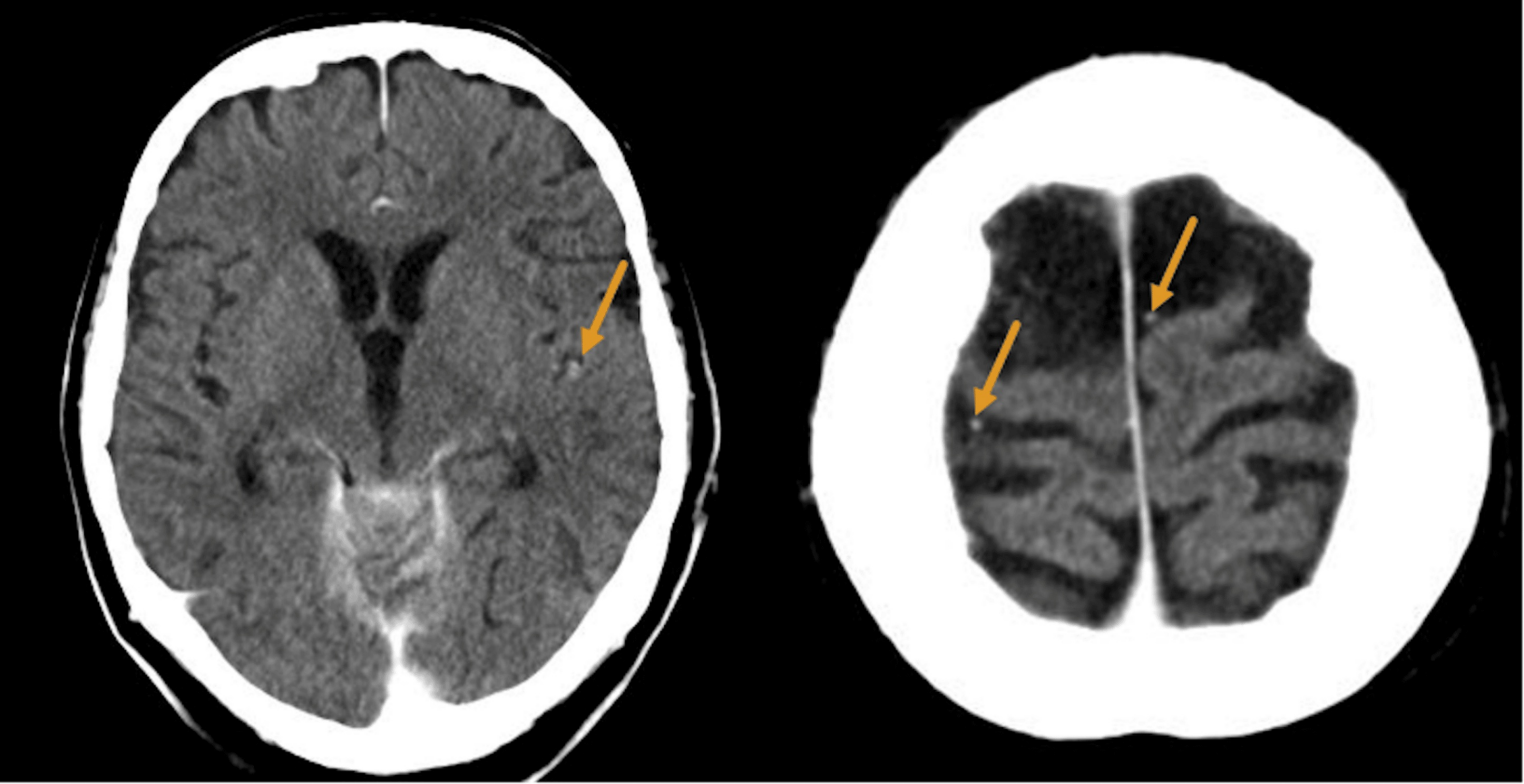
Brain computed tomography revealing suspected metastasis (arrows).

The patient remained hospitalized for approximately two weeks for further diagnostic evaluation, during which she developed a generalized tonic-clonic seizure. A computed tomography (CT) of the brain (Figure 8) was performed, revealing focal nodular characteristics along the brainstem-cerebellum and the temporal lobes bilaterally, possibly in the context of leptomeningeal dissemination. Confirmation should have been obtained with magnetic resonance imaging (MRI); however, this was not performed due to the patient's critical condition.

During hospitalization, the patient exhibited progressive clinical deterioration, characterized by worsening generalized weakness, reduced responsiveness, and impaired communication. Following initial evaluation, she was referred to an oncology center; however, due to her poor performance status, she was able to receive only limited systemic therapy. She was treated with dual anti-HER2-targeted therapy in October 2025, consisting of the monoclonal antibodies trastuzumab and pertuzumab, injected subcutaneously.

Her clinical condition continued to decline, and she subsequently developed severe pneumonia complicated by sepsis, leading to further deterioration and eventual clinical collapse in November 2025. A comprehensive timeline of the patient's clinical course is presented in Table 3.

**Table 3:** Timeline of the patient's clinical course. ER, estrogen receptor; PR, progesterone receptor; CT, computed tomography; IHC, immunohistochemistry; TIRADS, Thyroid Imaging Reporting and Data System

Time	Actions/Events	
March 2025	Onset of progressive voice changes	
March-September 2025	Multiple hospital visits; no acute pathology identified	
September 2025	Presentation at our hospital	Malaise, severe headaches, and persistent dysphonia; palpable cervical induration on examination
	Initial laboratory evaluation	Normal thyroid function tests; mild elevation of CA 15-3
	Imaging (thyroid ultrasound)	Suspicious for malignancy (TIRADS)
	Biopsy (core needle biopsy)	Confirmed malignant neoplastic infiltration
	IHC	Profile consistent with metastatic breast carcinoma (ER+, PR+, GATA3+, and HER2 3+)
	Systemic evaluation	No primary breast lesion detected; CT showed a large cervical mass
	Clinical course (during hospitalization)	Seizure and neurological deterioration
Late October 2025	Initiation of trastuzumab + pertuzumab	
November 2025	Clinical decline with sepsis leading to death	

## Discussion

The metastatic involvement of the thyroid gland has been well-documented in the pathology literature for several decades, including in seminal works such as those by Rosai et al. [8], and continues to represent an uncommon clinical entity, accounting for approximately 1%-3% of all thyroid malignancies [1,9]. Metastases of non-thyroid malignancies to the thyroid are more common in women than men (female-to-male ratio = 1.4:1) and usually occur in the advanced stages and are often (up to 80%) asynchronous, as in this case [1].

Occult breast cancer (OBC) is a rare clinical entity, defined as metastatic carcinoma consistent with breast origin in the absence of an identifiable primary breast lesion on clinical examination and conventional imaging. It most commonly presents with axillary lymph node involvement, whereas distant metastases as the initial manifestation are exceptionally rare [10-12]. In our case, no suspected axillary lymphadenopathy was detected, except for some benign-looking lymph nodes.

The thyroid gland represents an uncommon site of metastasis. The most frequent primary tumors metastasizing to the thyroid include renal cell carcinoma, lung carcinoma, and melanoma, while breast carcinoma represents a less common source [9,10]. However, metastases may occur metachronously, developing months or even years after the initial diagnosis of the primary tumor [13].

In the present case, thyroid involvement constituted the initial manifestation of OBC, a particularly unusual scenario that has been only rarely described in the literature [10,14]. This presentation underscores the diagnostic complexity of metastatic adenocarcinoma of unknown primary origin, especially when clinical breast examination and conventional imaging are negative, as demonstrated in our case [15].

The diagnosis of thyroid metastasis is often challenging because clinical and radiological findings may mimic those of primary thyroid malignancies. Ultrasonographic features such as hypoechoic nodules, irregular margins, and increased vascularity are nonspecific and do not reliably distinguish primary thyroid cancer from secondary involvement [1,10,16]. Most of the lesions are solitary than diffuse or multiple, heterogeneous, and hypoechoic [6,17].

Therefore, histological diagnosis remains essential. Although fine-needle aspiration is frequently used as an initial diagnostic tool for thyroid lesions, the histopathological evaluation of core needle biopsy material, together with immunohistochemical profiling, is particularly valuable when metastatic disease is suspected [16]. In this setting, ER, PR, mammaglobin, and GATA3 positivity strongly support breast origin, whereas negativity for thyroglobulin, PAX8, and thyroid transcription factor-1 (TTF-1) helps exclude a primary thyroid malignancy [1,16]. TTF-1 and PAX8 show nuclear expression in follicular thyroid cells, while thyroglobulin (TG) serves as a specific indicator of thyroid differentiation. In breast carcinoma, the most commonly applied immunohistochemical markers for prognostic evaluation and therapeutic guidance include ER, PR, HER2, and p53. The nuclear expression of GATA3 is helpful in distinguishing metastatic tumors of breast or urothelial origin from other types of malignancies. Additionally, when evaluating possible breast cancer metastases, markers such as GCDFP-15 and mammaglobin may also be used, although they are less sensitive compared to GATA3 [18]. Our case recapitulates the full spectrum of the aforementioned morphological and immunohistochemical findings.

An additional diagnostic challenge in this case was the absence of detectable breast lesions on physical examination, mammography, and breast ultrasonography. Although breast MRI has greater sensitivity in identifying occult breast tumors, it was not feasible in our patient due to limited cooperation, illustrating a real-world limitation in the diagnostic workup of OBC [15,19].

The presence of neurological symptoms and the subsequent identification of brain metastases indicated advanced systemic dissemination and an aggressive disease course. Brain metastases in breast cancer are generally associated with poor prognosis and reflect biologically aggressive disease [13,20].

Management strategies depend on the extent of disease and the patient's overall clinical status [13,20]. Surgical treatment may be considered in selected patients with isolated thyroid metastasis, either for symptom relief or for diagnostic confirmation.

However, in disseminated disease, systemic therapy is generally preferred [6,10,20]. In our patient, the coexistence of thyroid and brain metastases supported the decision for systemic oncologic management rather than local surgical treatment alone.

Overall, this case highlights the importance of including breast carcinoma in the differential diagnosis of metastatic adenocarcinoma involving the thyroid gland, particularly in women with negative conventional breast imaging and immunohistochemical findings suggestive of breast origin [1].

To our knowledge, the simultaneous presentation of OBC with both thyroid and brain metastases remains exceedingly rare [14,19]. In our case, the patient had no history of breast cancer or mass in her breast, and the diagnosis was established based on histopathological examination, so these statements suggest the rarity of our case. This case therefore adds to the existing literature and emphasizes the need for heightened clinical suspicion in atypical presentations.

## Conclusions

Occult breast cancer presenting with simultaneous thyroid and brain metastases represents an exceptionally rare and diagnostically demanding clinical scenario. This case highlights the importance of maintaining a high index of suspicion for metastatic disease when evaluating thyroid lesions with atypical features, even in the absence of a detectable primary breast tumor on clinical examination and conventional imaging, especially in women. In this context, negative immunohistochemical staining for markers indicative of primary thyroid malignancies should raise suspicion for a metastatic origin and prompt further evaluation with site-specific markers, including those associated with breast carcinoma. Immunohistochemical profiling is essential for the accurate determination of tumor origin.

The early recognition of such presentations is critical for guiding appropriate therapeutic decision-making, as they are typically associated and an unfavorable prognosis. The greater awareness of this rare presentation may improve diagnostic accuracy and clinical management.
